# Association of problematic usage of the internet with burnout, depression, insomnia and quality of life among Hungarian recreational esports players: a cross sectional study

**DOI:** 10.3389/fpubh.2025.1619810

**Published:** 2025-08-08

**Authors:** Andrea Feher, Eva Fejes, Gergely Feher, Lilla Horvath, Antal Tibold

**Affiliations:** ^1^Centre for Occupational Medicine, Medical School, University of Pécs, Pécs, Hungary; ^2^Szent Rafael Hospital, Zalaegerszeg, Hungary; ^3^Hospital of Komló, Komló, Hungary

**Keywords:** esports, internet addiction, insomnia, depression, burnout, quality of life, cross-sectional

## Abstract

**Background:**

While recreational esports are often considered a harmless hobby, problematic internet use (PUI) may be an overlooked risk factor for poor mental health outcomes.

**Objective:**

This study aimed to investigate the associations between PUI and depression, burnout, insomnia, and quality of life in adult Hungarian recreational esports players.

**Methods:**

A total of 2,313 participants completed validated questionnaires, including the Problematic Internet Use Questionnaire (PIUQ), the Maslach Burnout Inventory (MBI), the 9-item short form of the Beck Depression Inventory (BDI-SF), the Athens Insomnia Scale, and the EQ-5D quality of life instrument. Statistical analyses included Student’s t-test, chi-square test (*χ*^2^), Pearson’s correlation, and multivariate logistic regression. Model fit was evaluated using the Hosmer-Lemeshow test, Nagelkerke *R*^2^, and area under the receiver operating characteristic curve (AUC-ROC).

**Results:**

Excessive internet use, as indicated by the PIUQ, was detected in 19.9% of respondents. PUI was significantly associated with emotional exhaustion (OR = 1.10, 95% CI: 1.08–1.12, *p* < 0.001), moderate to severe depression (OR = 3.25, 95% CI: 2.55–4.14, *p* < 0.001), severe insomnia (OR = 2.75, 95% CI: 1.85–4.08, *p* < 0.001), and reduced self-sufficiency as measured by the EQ-5D (OR = 1.85, 95% CI: 1.45–2.36, *p* < 0.001). The final model demonstrated good fit (Hosmer-Lemeshow *p* = 0.21), strong explanatory power (Nagelkerke *R*^2^ = 0.42), and excellent discrimination (AUC = 0.86).

**Conclusion:**

Problematic internet use is significantly associated with multiple adverse mental health outcomes among recreational gamers. These findings suggest the need for increased awareness and preventive strategies targeting PUI in adult populations engaged in digital entertainment.

## Introduction

The term ‘esports’ (electronic sports) refers to competitive video gaming where professional players or teams compete in organized tournaments. These competitions can involve various game genres, including first-person shooters (FPS), real-time strategy (RTS), and multiplayer online battle arenas (MOBA). Esports events are often streamed online and attract large audiences, with some tournaments offering significant prize pools. Popular games in esports include *League of Legends*, *Dota 2*, *Counter-Strike*, and *Fortnite* (1, 42).

Recreational esports refers to casual or amateur competitive gaming, where players engage in structured video game competitions for fun rather than for professional careers or large financial rewards. Unlike professional esports players, recreational players focus on enjoyment, community participation, and skill improvement rather than high-stakes competition. This can include local or online tournaments for non-professional players, school or university esports clubs, corporate or workplace gaming leagues, or casual competitive play on platforms like Discord or in social gaming groups. Recreational esports help foster gaming communities, encourage teamwork, and provide a stepping stone for those interested in professional gaming (2).

Recreational esports were initially considered a healthy hobby, as they may reduce stress and pressure. Compared to professional players, recreational gamers do not experience intense pressure to win, and playing for fun makes gaming more enjoyable and sustainable. This type of play provides a structured but flexible way to enjoy gaming without the time commitment required for professional play. Gamers can step back from high-pressure environments while still engaging in competitive play; therefore, it may help maintain a healthy work-life balance (3). It may also enhance social interactions and community support, as playing in a recreational setting fosters friendships and a sense of community, and reduces isolation, which is a common issue among professional gamers (3, 4). Casual leagues and tournaments encourage teamwork and social bonding, which can improve mental wellbeing (3). Finally, without the constant pressure of long practice hours, recreational esports may promote a healthier approach to gaming (1, 42). However, recent studies have demonstrated potential harmful effects, as recreational esports may increase the prevalence and severity of mental health issues as well as problematic internet use (1, 4).

Problematic usage of the internet (PUI) refers to excessive or unhealthy internet use that negatively impacts an individual’s daily life, including mental health or general wellbeing. It is sometimes referred to as internet addiction or compulsive internet use and can affect work, school, relationships, and both mental and physical health (5). As no evidence-based classification exists (except for online gaming and gambling), the prevalence of PUI varies depending on the questionnaire used, age, country, and study population. Research suggests that up to 14% of the general population may be affected (1, 6). PUI rates tend to be higher among adolescents and young adults, ranging from 10 to 30%, as they are more likely to engage in gaming, social media, and various forms of online entertainment. Asian countries, particularly China and South Korea, report higher rates (up to 20%) due to affordable, widespread internet access and a strong gaming culture (7).

Psychological factors may play an important role in the development of PUI, but causality is not fully understood, as only cross-sectional studies exist and longitudinal data are lacking. There is a strong association between PUI and anxiety and depression, as individuals with mental health issues may use the internet to escape negative emotions. Low self-esteem, impulsivity, and poor self-control make it difficult to regulate time spent online, thereby increasing the risk of excessive use (8). Social and environmental factors should also be considered. A lack of real-life social connections (e.g., loneliness or social isolation) may lead individuals to rely on the internet for companionship. Peer influence to stay connected or game with friends, combined with a lack of offline activities, can contribute to excessive internet use. Overly permissive or neglectful parenting may also increase screen time in children and adolescents (9). The role of technology-related factors is likewise significant. Cheap and easy internet access enables excessive usage, and social media, gaming (including esports), and streaming platforms employ algorithms to prolong user engagement. Remote work, online education (especially during the recent pandemic), and digital entertainment have further blurred the lines between necessary and excessive use (10).

Burnout is considered an occupational phenomenon—it is a state of emotional, physical, and mental exhaustion caused by prolonged or excessive stress, often (but not exclusively) related to work or other demanding responsibilities such as parenting or schoolwork (11). Affected individuals feel overwhelmed, emotionally drained, and unable to keep up with ongoing demands. Common causes of burnout are usually work- or personal life-related, including financial problems, lack of rest, and insufficient self-care. Personality traits also play a key role, including perfectionism, poor stress management, neuroticism, and emotional instability (12, 13). Although burnout is not classified as a medical condition, it is associated with significant mental and somatic disorders such as insomnia, anxiety, depression, substance abuse, chronic pain syndromes, cardiovascular diseases, and even sudden death in younger individuals (<45 years) (14). Although neither PUI nor burnout is officially classified as a medical disorder, both are strongly linked to mental conditions such as anxiety, depression, and insomnia. While causality remains unclear, psychological research suggests these conditions may interact, forming a complex, self-reinforcing cycle (15–17).

Several companies provide recreational esports services as a potential way to prevent burnout and mental exhaustion, thereby reducing the risk of subsequent depression (18, 43). Online gaming is believed to contribute to stress relief and the formation of social connections, thereby improving quality of life (QoL). On the other hand, recent studies have found that it represents a double-edged sword, as esports can also be associated with increased risks of burnout, insomnia, and depression, as well as high rates of PUI (1, 4, 16). Thus, the association between online gaming and quality of life appears nuanced and may be either positive or negative, depending on factors such as level of engagement and underlying mental health status (19).

However, the vast majority of the aforementioned PUI studies focused on adolescents, and studies investigating adult populations—particularly recreational esports players—are almost entirely absent. We have previously observed increased rates of PUI among adult recreational esports players (1). One of the most widely cited models for explaining problematic internet use (PUI) is the Interaction of Person-Affect-Cognition-Execution (I-PACE) model, developed by Brand and colleagues. This framework posits that PUI emerges through dynamic interactions between individual predispositions (e.g., personality traits, psychopathology), affective responses (e.g., stress, anxiety), cognitive biases (e.g., attentional or coping styles), and executive functions (e.g., impulse control or decision-making capacity) (20). Within this model, excessive gaming or internet use is conceptualized as a maladaptive coping mechanism for dealing with emotional distress or unmet psychological needs. Over time, reinforcement mechanisms (e.g., immediate gratification, social rewards) contribute to the persistence of problematic use. This theory is particularly relevant for understanding how emotional exhaustion, depression, and insomnia may predispose individuals to compulsive online behavior (21).

The aim of our current research was to explore the potential associations between PUI and depression, insomnia, burnout, and quality of life, while accounting for multiple covariates within the same population.

## Patients and methods

This cross-sectional study, conducted using an online questionnaire, was carried out between March 2020 and August 2020. Data collection was performed using a non-probability sampling method. The study protocol and documentation were approved by the Ethics Committee of the University of Pécs (8434-PTE 2020), as previously published (1). Participants were recruited online via Twitch.com, in collaboration with Universum 8 Zrt and two influencers (1). Only individuals aged 18 years or older who provided informed consent prior to participation were eligible. Participation was entirely voluntary and anonymous.

Demographic variables collected included gender, age, family structure, number of children, education level, employment status, and average weekly working hours. Risk factors assessed included smoking, alcohol consumption, and drug use, categorized as relatively regular or not. Pre-existing medical conditions considered included a history of diabetes, hypertension, cardiovascular diseases, musculoskeletal pain, and depression. Additionally, daily internet usage was recorded.

Problematic Internet Use (PUI) was measured using the Problematic Internet Use Questionnaire (PIUQ), originally developed by Demetrovics and colleagues, and validated in Hungarian (22). This instrument contains 18 items grouped into three subscales: obsession, neglect, and control disorder. The obsession subscale assesses intrusive thoughts and withdrawal symptoms (e.g., anxiety, restlessness when offline). Example item: *“How often do you feel tense, irritated, or stressed if you cannot use the Internet as long as you would like?”* The neglect subscale evaluates disregard of responsibilities and basic needs, e.g., *“How often do you stay online when you’d rather be sleeping?”* The control disorder subscale measures difficulties in limiting internet use, e.g., *“How often do you say ‘just a few more minutes’ while online?”* Each item is rated on a 5-point Likert scale from 1 (never) to 5 (always). A total score above 41 indicates problematic internet use (1, 23).

Burnout was assessed using the Maslach Burnout Inventory (MBI), which comprises three subscales: emotional exhaustion (e.g., feeling drained from schoolwork), cynicism (detachment from academic tasks), and professional efficacy (sense of accomplishment in studies) (24). Items reflect stress experienced over the past 3 months, such as *“I feel exhausted at the end of a school day.”* Responses are rated on a 7-point Likert scale from 0 (never) to 6 (every day). High emotional exhaustion and cynicism scores combined with low professional efficacy indicate burnout (17). The questionnaire is copyright-protected and available in several languages, including Hungarian [Mind (25)].

Depressive symptoms were evaluated using the Beck Depression Inventory—Short Form (BDI-SF). This 9-item instrument assesses symptoms such as indecisiveness, social withdrawal, fatigue, sleep disturbances, pessimism, self-criticism, and lack of joy. Each item is rated on a 4-point Likert scale from 1 to 4 (26). Scores above 9suggest depression, with severity categorized as mild (10–18), moderate (19–25), or severe (≥26) (26, 27). The BDI-SF has been validated in the Hungarian language.

Sleep disturbances were assessed using the Athens Insomnia Scale (AIS), an 8-item instrument with 5 items targeting nocturnal symptoms and 3 targeting daytime functioning. Each item is scored on a 4-point Likert scale from 0 to 3. A total score >6 suggests insomnia, while scores >10 indicate clinically significant insomnia (28). The Hungarian version of the AIS was used (29).

Quality of life was measured using the EQ-5D, a validated and widely used self-report instrument that evaluates five dimensions of daily functioning: mobility, self-care, usual activities, pain/discomfort, and anxiety/depression. Each dimension is rated on a scale from 0 (no problem) to 5 (extreme problems). The EQ-5D is available in Hungarian and numerous other languages (30).

Based on PIUQ scores, participants were categorized into two groups: problematic internet users (PIU score >41) and normal (non-problematic) users (PIU score ≤41). Data are expressed as means ± standard deviations (SD). Student’s t-tests or chi-square tests were applied to compare the groups and identify statistically significant differences in measured parameters. To assess independent associations with problematic internet use, a binary logistic regression analysis was performed. Only variables that showed significance in univariate analysis were included in the final model. The analysis controlled for differences in risk factors and medication use. For each variable, odds ratios (OR) and 95% confidence intervals (CI) were calculated. All statistical analyses were performed using IBM SPSS Statistics, version 22.0 (IBM Corp., Armonk, NY, USA).

## Results

### Sample characteristics

A total of 2,313 recreational esports players participated in the online survey. The vast majority (92.4%) identified as male. Descriptive data on demographics, risk factors, comorbidities, and internet usage patterns are summarized in Supplementary Table 1 and have been previously published.

### Prevalence of problematic internet use (PUI)

Based on the Problematic Internet Use Questionnaire (PIUQ), 19.9% (*n* = 461) of participants met criteria for PUI. In univariate comparisons, PUI was more prevalent among individuals aged 18–25 years (*χ*^2^ = 4.77, *p* = 0.029), those who were single (*χ*^2^ = 5.41, *p* = 0.020), and those who reported having children (*p* < 0.05). Student status (*χ*^2^ = 4.64, *p* = 0.031), casual (*χ*^2^ = 4.64, *p* = 0.031) or secondary employment (*χ*^2^ = 4.36, *p* = 0.036), and working fewer than 10 h per week (*χ*^2^ = 4.44, *p* = 0.035) were also associated with increased odds of PUI. Moreover, alcohol consumption (*χ*^2^ = 9.81, *p* = 0.002), hypertension (*χ*^2^ = 9.95, *p* = 0.0018), and a history of depression (*χ*^2^ = 14.0, *p* < 0.01) were significantly more common among those with PUI (see Supplementary Table 2).

### Burnout symptoms

Based on the Maslach Burnout Inventory, 40.8% of participants had mild, 54.1% moderate, and 5.1% severe burnout (Table 1). Subscale means were: emotional exhaustion = 7.55 ± 6.7, depersonalization = 5.45 ± 5.9, and personal accomplishment = 19.5 ± 11.7. Emotional exhaustion levels were mild in 73.9%, moderate in 20.2%, and severe in 5.9%. Depersonalization levels were mild in 75.2%, moderate in 17.9%, and severe in 6.9%. Personal accomplishment scores indicated mild burnout in 34.4%, moderate in 28.1%, and severe in 37.6%.

**Table 1:** The association of PUI with burnout, depression and insomnia.

Category	Normal users (*n* = 1,852)	Problematic users (*n* = 461)	*χ* ^2^	*p*-value
Burnout				
Low	41.9% (775/1,852)	36.9% (170/461)	73.2	<0.001
Moderate	54.9% (1,017/1,852)	51.0% (235/461)		
Severe	3.2% (60/1,852)	12.1% (56/461)		
Depression				
No depression	29.0% (537/1,852)	9.7% (45/461)	305.6	<0.001
Mild	59.3% (1,099/1,852)	50.1% (231/461)		
Moderate	11.2% (207/1,852)	38.2% (176/461)		
Severe	0.5% (9/1,852)	2.0% (9/461)		
Sleep disturbance				
No	76.4% (1,415/1,852)	45.6% (210/461)	251.5	<0.001
Abnormal	19.7% (364/1,852)	41.2% (190/461)		
Severe	3.9% (73/1,852)	13.2% (61/461)		

PUI, problematic usage of the internet.

Participants with PUI showed significantly greater emotional exhaustion (11.43 ± 8.2 vs. 6.59 ± 5.9, *p* < 0.01, Cohen’s *d* = 0.68), higher depersonalization (8.76 ± 6.9 vs. 4.60 ± 5.3, *p* < 0.001, *d* = 0.70), and lower personal accomplishment (17.14 ± 10.3 vs. 20.0 ± 11.9, *p* < 0.001, *d* = 0.26). Overall, burnout was strongly associated with PUI (*χ*^2^ = 73.2, *p* < 0.001) (Table 2).

**Table 2:** The association of PUI with subcategories of burnout and QoL.

Variable	Normal users (*n* = 1,852)	Problematic users (*n* = 461)	*t*-statistic	*p*-value	Cohen’s *d*
Burnout					
Emotional exhaustion	6.59 ± 5.9	11.43 ± 8.2	−14.6	<0.001	0.68
Depersonalisation	4.6 ± 5.3	8.76 ± 6.9	−14.2	<0.001	0.70
Personal accomplishment	20.0 ± 11.9	17.14 ± 10.3	5.3	<0.001	0.26
Quality of life (points)	85.03 ± 14.99	78.2 ± 18.12	8.5	<0.001	0.42

PUI, problematic usage of the internet; QoL, quality of life.

### Depression and insomnia

Regarding depression, 25.2% of participants were symptom-free according to the Beck Depression Inventory (BDI), while 57.5% had mild, 16.6% moderate, and 0.7% severe symptoms. PUI prevalence increased with depression severity (*χ*^2^ = 305.6, *p* < 0.001). Insomnia symptoms were present in 23.9% of respondents, with an additional 5.8% reporting severe sleep disturbance. PUI was significantly associated with insomnia (*χ*^2^ = 251.5, *p* < 0.001).

### Quality of life

Participants with PUI reported lower scores on the EQ-5D Visual Analogue Scale (VAS): 78.2 ± 18.12 compared to 85.03 ± 14.99 in non-PUI individuals (*p* < 0.001). This difference was primarily driven by impairments in self-care and limitations in usual activities (*p* < 0.05 for both; data not shown).

### Multivariate analysis: predictors of PUI

A logistic regression model was constructed including variables significant in univariate analyses: age, relationship status, number of children, student status, employment type and hours worked, alcohol use, hypertension, history of depression, emotional exhaustion, depression (BDI), insomnia (AIS), and quality of life (EQ-5D) (Table 3).

**Table 3:** Results of the logistic regression analysis.

Predictor	aOR	95% CI	*p*-value
*Emotional Exhaustion* (per 1-point increase)	1.10	1.08–1.12	**<0.001**
*Moderate/Severe Depression* (vs. None/Mild)	3.25	2.55–4.14	**<0.001**
*Severe Sleep Disturbance* (vs. None)	2.75	1.85–4.08	**<0.001**
*Self sufficiency (EQ-5D)* (per 1-point increase)	1.85	1.45–2.36	**<0.001**

aOR, adjusted odds ratio; CI, confidence interval.

Bold values indicate statistically significant results (*p* < 0.05).

The model showed good explanatory power (Nagelkerke *R*^2^ = 0.42), adequate calibration (Hosmer–Lemeshow *p* = 0.21), and excellent discrimination (AUC = 0.86).

Independent predictors of PUI included:

- Emotional exhaustion (OR = 1.10 per point; 95% CI: 1.08–1.12; *p* < 0.001)
- Depression (OR = 3.25; 95% CI: 2.55–4.14; *p* < 0.001)
- Insomnia (OR = 2.75; 95% CI: 1.85–4.08; *p* < 0.001)
- Impaired self-sufficiency (OR = 1.85; 95% CI: 1.45–2.36; *p* < 0.001).

## Discussion

Our study is among the first to investigate the prevalence of problematic internet use (PUI) and its associated mental health correlates in recreational esports players. While recreational esports services are often promoted by companies for their potential benefits—including social bonding, cognitive stimulation, teamwork, and stress relief—our findings, along with recent studies, suggest that the relationship is complex and potentially bidirectional. Esports may not only provide stress relief but also be associated with increased risk of burnout, insomnia, depression, and PUI, highlighting a potential double-edged sword effect (1, 4, 16).

In our sample, the PUI prevalence was nearly 20%, which significantly exceeds the previously reported average of ~7% and is notably higher than the rates seen in other adult populations, such as teachers and healthcare workers (1, 17, 31). Prior studies have estimated that about 5% of the adult population may be affected by PUI; hence, our results underscore the possible link between online gaming/esports participation and compulsive internet use, warranting further investigation.

The overall burnout rate—with nearly 60% of participants exhibiting moderate to severe symptoms—was unexpectedly high, comparable to those reported in highly vulnerable populations such as healthcare professionals (32). Burnout was significantly associated with PUI in both univariate and multivariate analyses, with each dimension—emotional exhaustion, depersonalization, and reduced personal accomplishment—showing significant differences between problematic and non-problematic internet users. These findings are consistent with our earlier results in different adult samples (17, 31). However, due to the cross-sectional nature of the study, causal direction cannot be determined.

The relationship between burnout and PUI may be bidirectional: on the one hand, chronic stress and emotional exhaustion may increase vulnerability to maladaptive coping behaviors such as compulsive internet use; on the other hand, problematic internet engagement may exacerbate existing social and functional impairments, ultimately leading to burnout (8, 16, 31). Interestingly, emotional exhaustion—considered the primary component of burnout—was the only burnout subdimension significantly associated with PUI in multivariate models. Emotional instability, elevated anxiety, and poor communication skills may lead to increased social withdrawal, which has been linked to both PUI and burnout (33, 34).

PUI was also significantly associated with depression and insomnia, both in univariate and multivariate analyses. While the exact mechanism is unclear, prior research suggests that underlying psychopathologies such as anxiety and depressive symptoms may drive individuals toward excessive internet use as a form of self-medication. Conversely, PUI may itself contribute to the development or worsening of these psychiatric symptoms. Insomnia, for instance, may result from excessive screen time and dysregulated sleep cycles, as previously reported (17, 35). Additionally, the higher prevalence of moderate to severe depression among participants with PUI highlights the need for early identification and intervention. Given that depression is projected to become the leading global cause of disability by 2030 (36), these associations are clinically significant. Previous studies have also reported that PUI is linked to increased risk of suicidal ideation and behavior, further supporting its recognition as a legitimate psychiatric condition, rather than a benign behavioral phase (37).

Although burnout and depression share overlapping symptoms, recent moderation analyses have confirmed that they represent distinct constructs (38). Our multivariate model also confirmed that both conditions independently contribute to PUI risk, supporting the need to address them as separate yet interrelated issues.

Given the broad impact of PUI on mental health and daily functioning, its association with reduced quality of life (QoL) is not surprising. However, this relationship remains understudied. A recent meta-analysis of 18 studies found that PUI is associated with lower QoL, especially in domains related to psychological and physical wellbeing (39). In our study, reduced QoL was primarily driven by limited ability to perform daily activities and decreased self-sufficiency, both of which were also significantly associated with PUI in multivariate models. Excessive internet use can disrupt basic routines such as personal hygiene, meal preparation, housework, studying, or working, ultimately leading to functional decline. As individuals increasingly rely on digital environments for gratification or emotional regulation, their real-life efficacy may deteriorate, further reinforcing dependency and disengagement from offline life (40, 41).

Our findings align well with the I-PACE model of addictive behaviors, which emphasizes the interplay between emotional, cognitive, and executive processes in the development of problematic internet use (21). Specifically, emotional exhaustion—the strongest burnout predictor of PUI in our study—may reflect impaired emotional regulation, which the I-PACE framework identifies as a key risk factor. Similarly, depressive symptoms and sleep disturbances may act as affective triggers that drive individuals toward excessive internet use as a form of maladaptive self-regulation. The reinforcing nature of digital platforms—providing immediate distraction, social validation, or escapism—may further entrench these behaviors, particularly among individuals with low executive control or heightened impulsivity. Future longitudinal studies should investigate whether targeted interventions (e.g., emotion regulation training or cognitive-behavioral therapy) could disrupt this cycle.

## Limitations

This study has several limitations. First, the lack of standardized criteria for PUI complicates interpretation and generalization. Second, the study was conducted shortly before the first COVID-19 outbreak in Hungary, which may have influenced digital behavior patterns. Third, although over 2,300 recreational esports players participated, the sample is not representative of the general or adolescent population. The use of non-probability sampling may introduce selection bias. Fourth, all data were self-reported and no clinical interviews, physical exams, or validated medical records were available. Therefore, conditions like depression or insomnia were assessed through questionnaire-based screening, not formal diagnosis and no longitudinal follow-up was conducted to examine changes over time.

Importantly, data collection took place during the COVID-19 pandemic, which may have significantly contributed to the elevated prevalence rates observed in our study. Prolonged lockdowns, reduced social interaction, remote education, and restricted access to physical activities likely intensified reliance on digital entertainment, including esports gaming. These unique environmental stressors may have amplified symptoms of burnout, depression, and insomnia, particularly among young adults. Thus, caution is warranted when generalizing these results beyond the pandemic context, and future studies should consider comparing post-pandemic data to evaluate the persistence or attenuation of these effects.

## Conclusion

To our knowledge, this is one of the largest and most comprehensive studies in Hungary investigating PUI among recreational esports players. Our findings indicate a notably high prevalence of internet addiction, burnout, depression, and insomnia in this group, suggesting that recreational esports—often perceived as a harmless or even beneficial activity—may carry significant mental health risks in certain subpopulations. The strong associations between PUI and various mental health dimensions underscore the need for screening, prevention, and targeted interventions, especially in digitally engaged young adults. These results may contribute to shifting the current narrative around recreational gaming and encourage the clinical recognition of PUI as a meaningful public health concern.

## Implications for future research and practice

Our findings highlight the need for more targeted interventions addressing problematic internet use (PUI) and its psychological correlates within recreational gaming populations. Future research should employ longitudinal designs to examine causal pathways and assess the directionality of the observed associations. Moreover, studies should incorporate neurocognitive assessments and ecological momentary methods to better understand the dynamic interplay between emotional exhaustion, depressive symptoms, sleep disturbances, and internet use patterns. In clinical and public health contexts, these results support the integration of mental health screenings into gaming-related platforms, the development of digital literacy and coping skills programs, and the implementation of personalized prevention strategies. A particular emphasis should be placed on at-risk subgroups, such as young adults with high emotional exhaustion and comorbid depressive or sleep disorders.

## Data Availability

The raw data supporting the conclusions of this article will be made available by the authors without undue reservation.
