# Supplementary material for: Association of problematic usage of the internet with burnout, depression, insomnia and quality of life among Hungarian recreational esports players: a cross sectional study
Source: Front Public Health. 2025 Aug 8;13:1619810. doi: 10.3389/fpubh.2025.1619810 (PMC12370677; doi:10.3389/fpubh.2025.1619810)
Supplement: Supplementary file 1 [file Table_1.docx]

| (N=2313) | % | |
| --- | --- | --- |
| **Gender** |  | |
| Female | 7.6 (176) | |
| Male | 92.4 (2137) | |
| **Age** |  | |
| 18-25 years | 90.3 (2088) | |
| 26-35 years | 7.95 (184) | |
| 36-45 years | 0.86 (20) | |
| 46-55 years | 0.82 (19) | |
| 56-62 years | 0.04 (1) | |
| above 62 years | 0.04 (1) | |
| **Marital status** |  | |
| single | 71.0 (1643) | |
| in relationship | 25.9 (600) | |
| married | 2.6 (59) | |
| divorced / widow | 0.5 (11) | |
| **Number of children** |  | |
| no children | 97.2 (2249) | |
| 1 child | 1.3 (30) | |
| 2 children | 1.1 (25) | |
| more than 3 children | 0.4 (9) | |
| **Eduactional background** |  | |
| elementary education | 30.1 (696) | |
| secondary education | 60.0 (1388) | |
| higher education | 9.9 (229) | |
| **Employment status** |  | |
| employment | 34.8 (804) | |
| entrepreneur | 3.5 (82) | |
| student | 30.1 (696) | |
| other | 31.6 (731) | |
| **Work shedule** |  | |
| full time | 35.4 (819) | |
| part time | 14.1 (326) | |
| flexible | 13.5 (313) | |
| other | 37.0 (855) | |
| **Time spent with work** |  | |
| less than 10 hours | 36.4 (842) | |
| 10-20 hours | 13.3 (308) | |
| 20-30 hours | 9.7 (225) | |
| 30-40 hours | 18.1 (419) | |
| more than 40 hours | 22.4 (519) | |
| **Secondary employment** |  | |
| no | 79.7 (1843) | |
| yes | 20.3 (470) | |
| **Concomitant diseases (%)** | |  |
| on regular medication | | 11.6 (268/2313) |
| smoking | | 25.3 (586/2313) |
| alcohol use | | 14.1 (325/2313) |
| drug use | | 22.0 (508/2313) |
| diabetes | | 2.3 (54/2313) |
| hypertension | | 8.5 (197/2313) |
| cardiovascular disease | | 3.6 (83/2313) |
| musculosceletal pain | | 2.6 (59/2313) |
| history of depression | | 2.9 (68/2313) |

**Table 1. Characteristics of the study population**.
