# Supplementary material for: Association of problematic usage of the internet with burnout, depression, insomnia and quality of life among Hungarian recreational esports players: a cross sectional study
Source: Front Public Health. 2025 Aug 8;13:1619810. doi: 10.3389/fpubh.2025.1619810 (PMC12370677; doi:10.3389/fpubh.2025.1619810)
Supplement: Supplementary file 2 [file Table_2.docx]

|  | | **Not addicted to internet (n= 1852)** | | **Internet addiction (n= 461)** |
| --- | --- | --- | --- | --- |
| **Gender** | | | | |
| Male | | 92.7% (1717/1852) | | 91.1% (420/461) |
| Female | | 7.3 % (135/1852) | | 8.9% (41/461) |
| **Age (years)** | | | | |
| ***18-25 years*** | | ***89.5% (1658/1852)*** | | ***93.3 % (430/461)**** |
| 26-35 years | | 8.4 % (155/1852) | | 6.3% (29/461) |
| 36-45 years | | 0.9 % (18/1852) | | 0.4 % (2/461) |
| 46-55 years | | 1.0 % (19/1852) | | 0% (0/461) |
| 56-62 years | | 0.1 % (1/1852) | | 0% (0/461) |
| above 62 years | | 0.1 % (1/1852) | | 0% (0/461) |
| **Marital status (%)** | | | | |
| ***single*** | | ***70.3% (1302/1852)*** | | ***74.0 % (341/461)**** |
| in relationship | | 26.3% (488/1852) | | 24.3 % (112/461) |
| married | | 3.0% (52/1852) | | 1.5% (7/461) |
| divorced / widow | | 0.4% (8/1852) | | 0.2% (1/461) |
| **Number of children** | | | | |
| no children | | 96.8% (1792/1852) | | 99.1% (457/461) |
| ***1 child*** | | ***1.5% (28/1852)*** | | ***4.5% (2/461)**** |
| ***2 children*** | | ***1.2% (23/1852)*** | | ***4.5% (2/461)**** |
| more than 3 children | | 0.5% (9/1852) | | 0% (0/461) |
| **Educational background** | | | | |
| elementary education | | 29.4% (545/1852) | | 32.8% (151/461) |
| secondary education | | 60.6% (1122/1852) | | 57.7% (266/461) |
| higher education | | 10.% (185/1852) | | 9.5% (44/461) |
| **Employment status** | |  | |  |
| employment | | 36.8% (682/1852) | | 26.5% (122/461) |
| entrepreneur | | 3.6% (67/1852) | | 3.3% (15/461) |
| ***student*** | | ***29.1% (539/1852)*** | | ***34.1% (157/461)**** |
| ***other*** | | ***30.5% (564/1852)*** | | ***36.1% (167/461)**** |
| **Work schedule** | |  | |  |
| full time | | 37.0% (686/1852) | | 28.8% (133/461) |
| part time | | 13.7% (253/1852) | | 15.8% (73/461) |
| flexible | | 13.3% (246/1852) | | 14.5% (67/461) |
| other | | 36.0% (667/1852) | | 40.9% (188/461) |
| **Time spent with work** | |  | |  |
| ***less than 10 hours*** | | ***35.4% (655/1852)*** | | ***40.6% (187/461)**** |
| 10-20 hours | | 13.0% (240/1852) | | 14.8% (68/461) |
| 20-30 hours | | 9.9% (183/1852) | | 9.1% (42/461) |
| 30-40 hours | | 18.6% (344/1852) | | 16.3% (75/461) |
| more than 40 hours | | 23.1% (430/1852) | | 19.2% (89/461) |
| **Secondary employment** | | | | |
| no | | 80.7% (1494/1852) | | 75.7% (349/461) |
| ***yes*** | | ***19.3% (358/1852)*** | | ***24.3% (112/461)**** |
| **Concomitant diseases** | | |  | |
| medication use | 11.4% (212/1852) | | 12.1% (56/461) | |
| current smoker | 26.1% (484/1852) | | 22.1% (102/461) | |
| ***alcohol use*** | ***12.8% (237/1852)*** | | ***19.1% (88/461)­**** | |
| drug use | 21.7% (402/1852) | | 22.9% (106/461) | |
| diabetes | 2.2% (40/1852) | | 3.0% (14/461) | |
| ***hypertension*** | ***7.5% (138/1852)*** | | ***12.8% (59/461)**** | |
| cardiovascular disease | 3.8% (70/1852) | | 4.9% (23/461) | |
| musculoskeletal pain | 2.4% (44/1852) | | 3.2% (15/461) | |
| ***history of depression*** | ***2.3% (43/1852)*** | | ***5.4% (25/461)***** | |
| **Daily internet use (approximately)** | | | | |
| 1 hour | 2.8% (52/1852) | | 0.9% (6/461) | |
| 2 hours | 15.1% (279/1852) | | 9.3% (43/461) | |
| 3 hours | 23.2% (430/1852) | | 16.1% (74/461) | |
| 4 hours | 22.7% (421/1852) | | 22.1% (102/461) | |
| 5 hours | 15.6% (289/1852) | | 18.4% (85/461) | |
| ***6 hours*** | ***6.7% (124/1852)*** | | ***9.5% (44/461)**** | |
| ***> 6 hours*** | ***13.9% (260/1852)*** | | ***23.2% (107/461)***** | |

**Table 2. Comparison of baseline characteristics of the study subgroups (*p<0.05; **p<0.001).**
